# Influence of Bone Conduction Hearing Device Implantation on Health-Related Quality of Life for Patients with and without Tinnitus

**DOI:** 10.3390/audiolres13040050

**Published:** 2023-08-01

**Authors:** Nasrene Khan, Aaran T. Lewis

**Affiliations:** 1 School of Public Health and Community Medicine, University of Gothenburg, 40530 Gothenburg, Sweden; gusnasrkh@student.gu.se; 2 Cochlear Limited, 43533 Mölnlycke, Sweden

**Keywords:** hearing loss, hearing rehabilitation, tinnitus, bone conduction implant, quality of life, utility gain

## Abstract

(1) Background: Tinnitus, often related to hearing loss, is an addressable public health concern affecting health-related quality of life (HRQoL). This study aimed to explore the influence of bone conduction hearing aid (BCHA) implantation on HRQoL and hearing disability in patients with hearing loss suffering from tinnitus. (2) Methods: Data were collected from an international hearing implant registry. Health Utilities Index Mark 3 (HUI-3), Spatial and Qualities of Hearing- 49 Questionnaire (SSQ) and self-reported tinnitus burden data for adult patients implanted with a BCHA (n = 42) who provided baseline as well as follow-up data 1-year post-implantation were extracted from the registry. Wilcoxon signed rank tests and paired samples t-tests were used to analyse outcomes data. (3) Results: Patients, with or without tinnitus, demonstrated clinically important mean improvements in HUI-3 multi-attribute utility scores, HUI-3 hearing attribute and SSQ scores. Hearing loss patients with tinnitus presented with a lower HRQoL than patients without tinnitus. (4) Conclusions: These findings demonstrate the importance of hearing rehabilitation in improving the quality of life and hearing disability of patients with or without tinnitus and in providing tinnitus relief in some patients with hearing loss and tinnitus.

## 1. Introduction

The term tinnitus describes a perceived auditory sensation in the absence of an associated external auditory stimulus [1]. Tinnitus is experienced with varying intensity as a buzzing or ringing sound or sometimes as rhythmical or pulsatile [1,2]. It can affect those with normal hearing [3]; however, it is also associated with many other conditions, including noise-induced hearing loss, ear infections and acoustic trauma [4]. The prevalence of tinnitus varies due to the subjective nature of the disease as its severity is usually determined by the level of worry or concern expressed by the patient [5]. Some studies have reported a range of 10–15% [4,6,7] of the world’s general adult population being affected, whereby only a quarter of these persons seek medical help [4].

Tinnitus is often directly related to age and hearing loss [8]. However, other predisposing factors such as injuries to the head and neck, ototoxic drug use, infections and a wide range of other medical indications can contribute to the disease [9]. Whilst many with tinnitus have mild symptoms and are able to cope with the disease, some patients experience severe tinnitus which can be unbearable and severely impactful on their quality of life [10]. Persons who are exposed to both stress and noise have an increased probability of exacerbating their symptoms [7]. In addition, tinnitus is closely related to stress, anxiety, depression, sleep deprivation and decreased work productivity [2,11,12], whereby one study reported that 60% of tinnitus patients have major depression [13]. The fear and anxiety associated with the tinnitus symptoms itself can lead to an ongoing cycle involving mental illness and tinnitus severity [14]. Another study reported reduced health-related quality of life (HRQoL), general anxiousness and perceived handicap in 40–50% of patients with tinnitus [15].

Currently, there is no cure for tinnitus; however, non-standardised interventions with varying efficacies exist for symptomatic relief, including auditory stimulation, education and reassurance, and cognitive behavioural therapy (CBT) [16]. Psychotherapeutic methods such as CBT for tinnitus management remain contentious as many patients find it unsuitable to manage their symptoms since they characterize them as somatic rather than psychological [17]. The available treatment options for tinnitus are, therefore, suboptimal as they only reduce the disease severity and may result in a financial burden for the patient [18]. Despite its prominent socioeconomic impact, economic evaluations on tinnitus are scarce and limited resources are invested in tinnitus research [19].

Tinnitus is a severe condition comparable to other major chronic diseases [20]. Its high prevalence, non-standardised treatment options and complex association with mental and physical health, socioeconomic burden, and overall quality of life make tinnitus an addressable public health issue [12,18,21]. It is common for tinnitus to coexist with a hearing deficit [8] and 85% of tinnitus cases are accompanied by hearing loss [22]. Hearing loss is a global health burden affecting more than 1.5 billion people and imposes a global cost of USD 980 billion annually [23]. Hearing loss, tinnitus, and related hearing disorders should be addressed to optimally manage the disease [12] and ameliorate the global health burden in line with the United Nations 2030 health goals.

According to the literature, some tinnitus sufferers have reported worsening symptoms in noisy areas; however, many have reported tinnitus aggravation from a lack of background noise, whereby its addition helped suppress their tinnitus [24,25]. Tinnitus relief in some patients can occur when external auditory loss is restored [2]. Acoustic implants (AI), such as bone conduction hearing aids (BCHA), transmit sound energy from vibrations in the skull to the cochlea to stimulate hearing [26]. They aim to treat patients suffering from significant conductive or mixed hearing loss who would benefit from sound amplification but are unable to wear air-conduction hearing aids [27]. There is limited published data on BCHA and tinnitus; however, a recent study showed promising results post-implantation with an active transcutaneous BCHA in the treatment of tinnitus caused by unilateral sensorineural hearing loss [28].

Subjective data on patient-reported outcomes using standardised measures on HRQoL such as the disease-specific Speech, Spatial and Qualities of Hearing Scale (SSQ) and the Health Utilities Index Mark 3 (HUI-3) are necessitated in addition to objective clinical outcome measures for optimal disease management [29]. SSQ is a reliable, self-reported questionnaire for adults based on their day-to-day hearing performance, divided into three categories, including 14 scored items on speech hearing; 17 items on spatial hearing; and 19 items on other functions and qualities of hearing. SSQ provides invaluable insight into what influences hearing handicaps [29,30] and can be administered to patients following treatment with a variety of hearing devices [31]. HUI-3 is a standardised, valid and widely recommended tool used to comprehensively measure HRQoL when evaluating health interventions [32]. HUI-3 uses single- and multi-attribute utility functions to measure health status and its classification system comprises eight attributes including vision, hearing, speech, ambulation, dexterity, emotion, cognition and pain [33]. The HUI-3 measure of HRQoL ranges between 0 and 1 where a 0 utility equates to death and a utility assignment of 1 equates to a perfect health state [34]. HUI-3 and SSQ allow for the quantification of the outcomes associated with the disease as well as the effectiveness of the possible tinnitus intervention [30,34]. They can be used to determine quality-adjusted life years (QALY), which would support clinical evaluations and economic modelling of tinnitus management interventions [35]. Available studies based on these measures have methodological shortcomings [19] since they are low-powered or use legacy devices, and information on the device’s impact on HRQoL for patients with tinnitus is minimal [18].

Long-term follow-up based on acoustic hearing implantation influence on HRQoL in tinnitus patients are not readily available in the literature. Furthermore, these studies suffer from limited sample size and non-standardised reporting [36,37]. This study aims to explore the impact of BCHA implants on HRQoL and hearing disability in patients suffering from tinnitus.

## 2. Methods

### 2.1. Study Design

The study utilised retrospective data collected during the Cochlear Implant Recipient Observational Study (IROS). IROS was a prospective, longitudinal study that used repeated measures with intra-subject controls. The study used objective audiological measures and subjective evaluation tools such as the SSQ and HUI-3 to evaluate HRQoL and patient-related benefits of the use of implantable hearing devices, including BCHA devices. 

The IROS is an international registry that also collected baseline and follow-up socio-economic information on adult recipients, including information on tinnitus burden. IROS is registered on ClinicalTrials.gov with the identifier NCT02004353. The design, implementation and management of the registry have been published previously [38]. The registry was active from 2011 to mid-2020 and included 77 clinics globally with accessibility to these devices and with adequate infrastructure required for data collection and resources to provide long-term follow-up. Participation in the registry was voluntary by clinics and patients.

The presence of tinnitus, defined as noise or ringing in the head or ears, was assessed using a non-standardised form within the IROS patient questionnaire. Subjects were first asked to report pre-implantation if they experienced tinnitus. If yes, they were asked to complete follow-up questions based on how often it was experienced: always; sometimes; and do not know. The same questions were asked post-surgery, at the 1-year follow-up. Thereafter, a single question was asked based on how subjects would describe their tinnitus in comparison to pre-implantation: worse; same; better; and do not know. 

**Figure 1:**
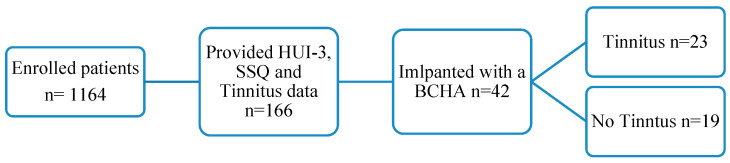
Patient eligibility and inclusion flow chart at 1-year follow-up.

The registry contains baseline data, pre-implantation, and follow-up data for up to 3 years post-implantation, although there is significant attrition after 1-year follow-up. A total of 1164 subjects that received an implantable hearing device provided baseline data, but only 180 recipients provided data at the three-year follow-up visit (Y1—n: 647; Y2—n: 337; Y3—n: 180). The study included all eligible adult patients receiving a BCHA device who have provided baseline as well as follow-up data after 1 year, including information on HUI-3, SSQ and self-reported tinnitus burden (Figure 1). Eligible data collected at any time during the entire 10.5-year period were included in the study. 

### 2.2. Ethical Considerations

The IROS study was approved by the Ethical Review Boards of participating clinics in Colombia (Clínica Rivas, CEL 5277), Germany (Medizinische Hochschule Hannover, 1241–2011), Hungary (Egészségügyi Nyilvántartási és Képzési Központ, 070662/2015/OTIG), Poland (Uniwersytetu Medycznego w Łodzi, RNN/117/12/KE), Spain (Hospital de la SantaCreu i Sant Pau, HSCSP 11/083), and South Africa (Stellenbosch University, N15/02/015) according to institutional and national research standards. All patients provided written informed consent prior to inclusion.

### 2.3. Statistical Analysis

Data were analysed using IBM SPSS version 29. Socio-demographic and other relevant clinical and nonclinical categorical data, including background characteristics at baseline, are presented descriptively. Categoric variables are represented as a number (n) and/or percentage. Continuous background data are presented as a mean value with standard deviation (SD).

HUI-3 questionnaire data were analysed, and attribute and utility scores were calculated following standard methodology: Calculation Matrix HUI-3 Multi-attribute Utility HUI3 Multi-attribute Utility Function on Dead-healthy Scale Matrix [39].

SSQ questionnaire scores from each domain were combined into mean domain-specific scores and a global mean score by taking the average score across each scale. HUI-3 and SSQ data were assessed graphically and by Kolmogorov–Smirnov and Shapiro–Wilk tests. These tests confirmed unequal distribution of the HUI-3 overall utility and hearing attribute scores pre- and post-implantation and normal distribution for the SSQ scores pre- and post-implantation. Therefore, the parametric Wilcoxon signed rank test for related samples was applied to determine mean utility and hearing attribute changes and paired t-tests were used for the SSQ score differences. A *p*-value of *p* < 0.05 was used to determine statistical significance.

Effect size (d) values are presented as an additional measure to demonstrate the magnitude of the treatment effect, independent of sample size [40]. Effect size values around d = 0.2 are considered small; around d = 0.5 are considered medium; and d ≥ 0.8 are considered a large effect size [41]. The use of effect size and clinically meaningful measures to complement *p*-values are widely encouraged to enhance research quality [42]. 

Point-biserial correlation tests were conducted to measure the strength of the association between tinnitus status and change in HUI-3 and SSQ scores pre- and post-implantation. Standard correlation value guidelines were applied whereby coefficient values between 0 and ±0.3 indicated a weak linear relationship, values between ±0.3 and ±0.7 indicate a moderate linear relationship, and values between ±0.7 and ±1 indicate a strong relationship. The positive or negative value indicates the direction of the strength of the relationship [43]. 

HUI-3 mean overall HRQoL score differences of 0.03 or greater are considered clinically important, and differences of at least 0.01 may be meaningful and important in some contexts [44]. Differences in hearing disability SSQ scores of ≥1 unit are considered clinically relevant [30].

## 3. Results

### 3.1. Demographics and Statistics Summary

#### Study Sample

The study included 42 IROS participants who were implanted with a BCHA device. Forty patients received a transcutaneous device and the remaining 2 patients received percutaneous devices. The presence of tinnitus was reported by 23 participants at baseline, and 19 participants presented without tinnitus at baseline. IROS participants who had HUI-3 and SSQ data at baseline and one-year post-implantation were included. Participants who answered “do not know” for tinnitus-related questions at baseline or had missing tinnitus status data were excluded from the study.

The study comprised about 60% males and 40% females. The mean age of the group was 40.21 (±14.58) years, and a majority of the participant group was based in Columbia (85.7%), with the remainder based in Poland (14.3%) (Table 1). 

### 3.2. Bone Conduction Hearing Aid Scores

**Figure 2:**
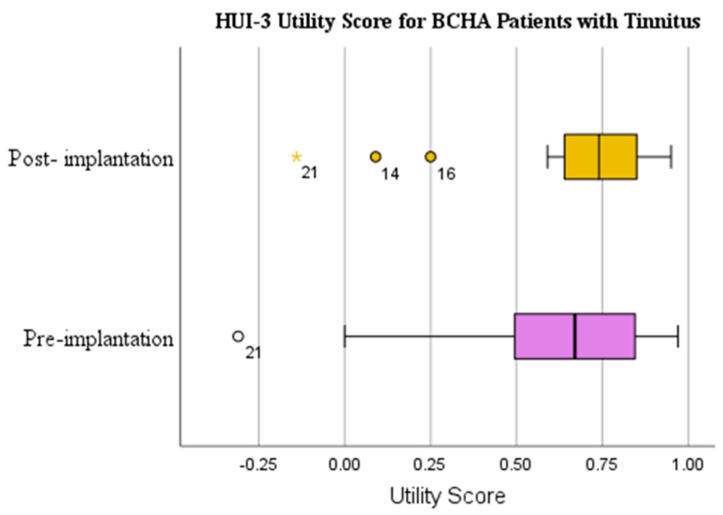
HUI3 utility scores pre-implantation and at 1-year post-implantation for BCHA patients with tinnitus. Circles represent outliers.

**Figure 3:**
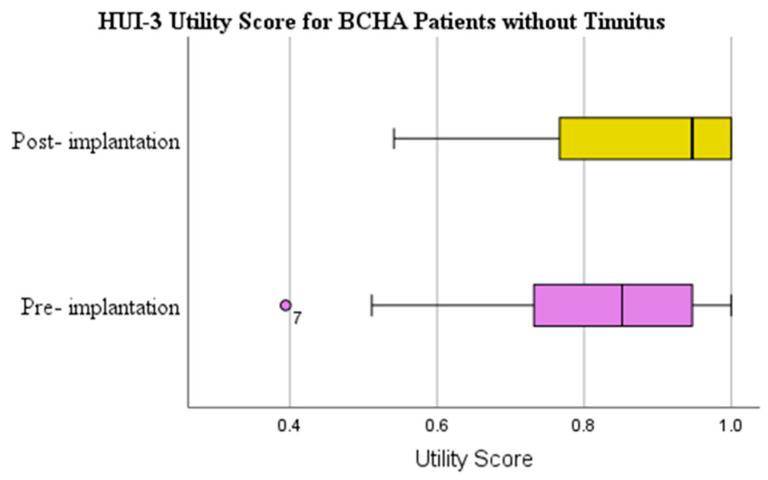
HUI-3 utility scores pre-implantation and at 1-year post-implantation for BCHA patients without tinnitus. Circles represent outliers.

The HUI-3 mean scores for patients with tinnitus showed a clinically important mean improvement of 0.054 from pre-implantation (0.624) to post-implantation (0.678) (Table 2 and Figure 2). The Wilcoxon signed rank test demonstrated that the results were not statistically significant (*p* = 0.218) and had a small effect size (d = 0.182). The HUI-3 mean utility score change for patients without tinnitus presented a clinically important mean improvement of 0.065 from pre-implantation (0.811) to post-implantation (0.876) (Figure 3); however, the Wilcoxon signed rank test showed that the results were not statistically significant (*p* = 0.277) and had a small effect size (d = 0.176).

#### 3.2.1. Health Utilities Index Mark 3: Hearing

**Figure 4:**
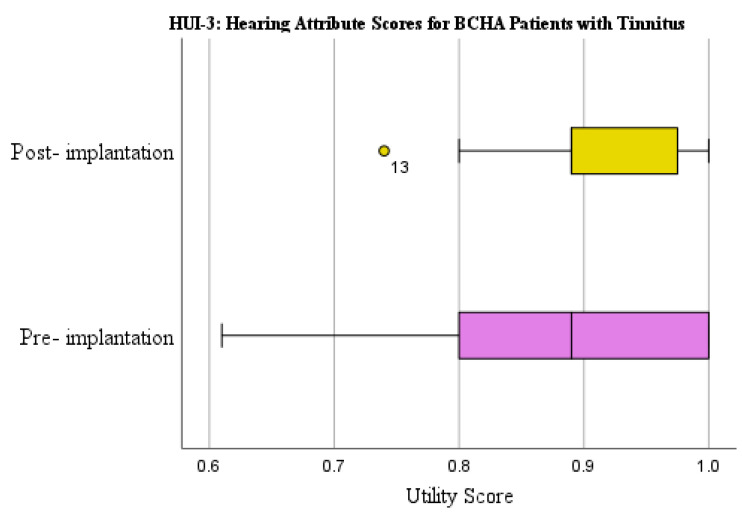
HUI-3 hearing attribute scores pre-implantation and at 1-year post-implantation for BCHA patients with tinnitus. Circles represent outliers.

The HUI-3 hearing attribute mean score change for patients with tinnitus demonstrated a clinically relevant improvement of 0.051 from pre-implantation (0.865) to post-implantation (0.916) (Figure 4). Results were however not statistically significant (*p* = 0.139) and had a small effect size (d = 0.218). 

**Figure 5:**
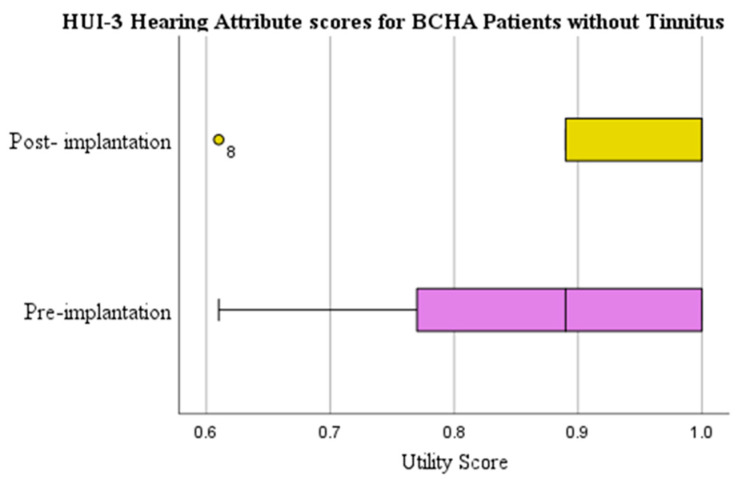
HUI-3 hearing attribute scores pre-implantation and at 1-year post-implantation for BCHA patients without tinnitus. Circles represent outliers.

The HUI-3 hearing attribute mean score change for patients without tinnitus presented a clinically relevant improvement of 0.076 from pre-implantation (0.857) to post-implantation (0.933) (Figure 5). Results were, however, also not statistically significant (*p* = 0.053) but displayed a medium effect size (d = 0.313).

#### 3.2.2. Speech, Spatial and Qualities of Hearing Scale-49 

**Figure 6:**
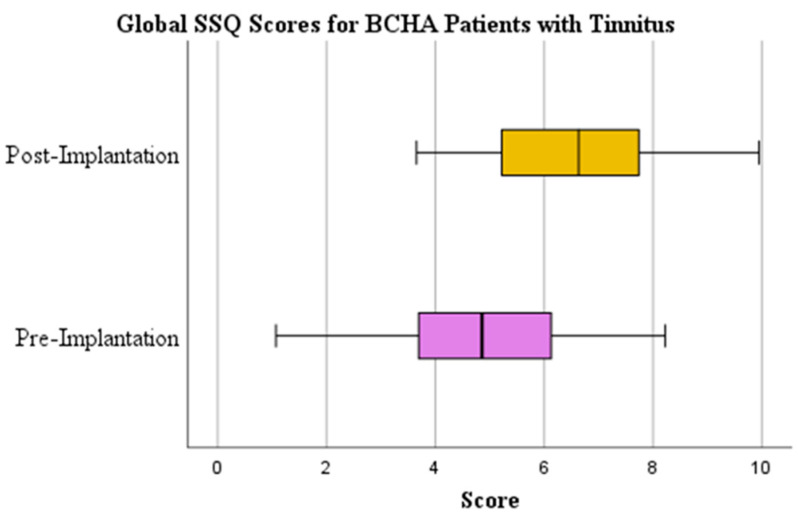
Global SSQ scores pre-implantation and at 1-year post-implantation for BCHA patients with tinnitus.

**Figure 7:**
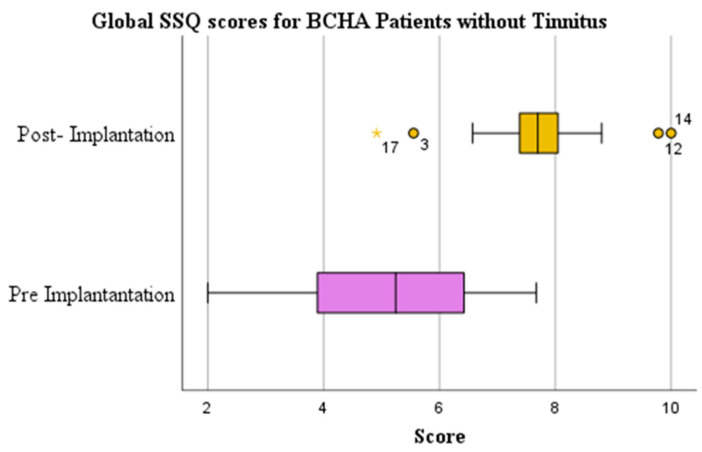
Global SSQ scores pre-implantation and at 1-year post-implantation for BCHA patients without tinnitus. Circles and asterisks represent outliers.

Results from the paired t-test in Table 1 above show a statistically significant (*p* < 0.001) and clinically relevant global SSQ mean improvement from pre (4.794) to post-implantation (6.566) for patients with tinnitus of 1.772 (Figure 6) and displayed a large effect size (d = 0.923). There was a statistically significant (*p* < 0.001) and clinically relevant global SSQ mean improvement from pre (5.036) to post-implantation (7.684) in patients without tinnitus of 2.648 (Figure 7) and the effect size was large (d = 1.445).

### 3.3. Tinnitus Perception

**Figure 8:**
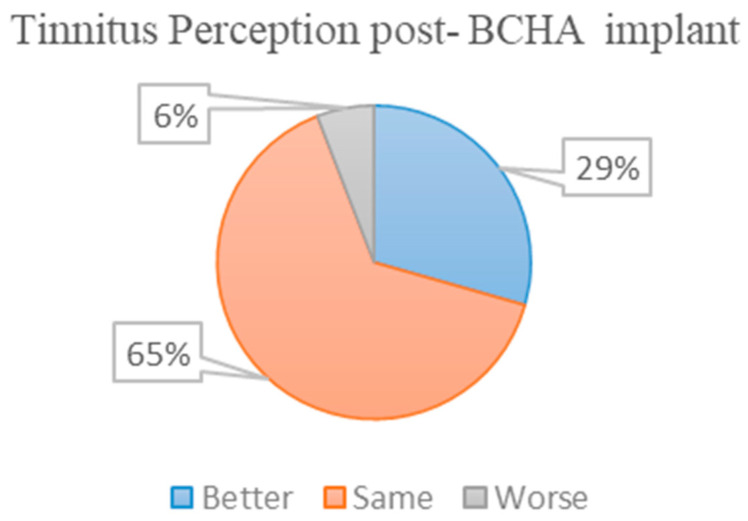
A pie chart demonstrating tinnitus perception post-implantation based on the self-reported questionnaire results.

Figure 8 demonstrates that of the 23 patients with tinnitus, 17 patients were able to confidently rate their tinnitus status 1-year post-implantation; 29% of these patients reported an improvement in their tinnitus symptoms; 65% reported no change in tinnitus symptoms and 6% reported worse symptoms post-implantation.

Results show that there was a statistically significant difference in pre-operative HUI-3 score depending on tinnitus status in patients that were later implanted with a BCHA (*p* = 0.026) (Table 3), which was moderately negatively correlated (r = −0.343). There was, however, no correlation between tinnitus status and change in HUI-3 scores post-implantation (r = −0.019, *p* = 0.906). No other comparisons were statistically significant.

## 4. Discussion

The aim of this study was to explore the impact of BCHA on HRQoL and hearing disability in patients suffering from tinnitus by using standardised instruments in the form of HUI-3 and SSQ questionnaires. Subjective hearing assessments allow for a more comprehensive understanding of audiometrically assessed handicaps and provide information based on hearing ability pre- and post-intervention as well as additional hearing-related effects and its psychosocial impact [45,46]. The international IROS registry uses widely accepted, cross-culturally adapted questionnaires such as HUI-3 and SSQ that enable a deeper real-world understanding of patient-related hearing benefits [38].

Important findings obtained from the HUI-3 measurements were that there were clinically relevant improvements in mean utility scores from pre- to post-implantation. The overall utility change (0.054, *p* = 0.218) and hearing attribute scores (0.051, *p* = 0.139) for tinnitus patients and the overall utility change (0.065, *p* = 0.277) and hearing attribute scores (0.076, *p* = 0.053) for patients without tinnitus, however, yielded differences not reaching statistical significance, likely due to the small sample size. The mean score improvements between tinnitus (0.054) and non-tinnitus (0.065) patients are similar post-implantation and the correlation results show that there are no significant associations between tinnitus status and change in HUI-3 scores post-BCHA-implantation (r = −0.019, *p* = 0.906). This indicates that baseline tinnitus status is likely to have a negligible impact on change in HUI-3 multi-attribute scores between pre- and post-device implantation. Tinnitus status and pre-implant HUI-3 scores, however, are significantly negatively correlated (r = −0.343, *p* = 0.026). This indicates that patients with tinnitus are likely to present with lower HUI-3 scores before treatment than patients without tinnitus. This is also expected as individuals with untreated hearing loss without tinnitus have been shown to experience greater HRQoL than those with both untreated tinnitus and hearing loss [47].

The standardised SSQ system provides detailed information focused on patients’ auditory performance in everyday situations to further enhance the data analysis [45]. There were clinically relevant and statistically significant mean SSQ score improvements for patients with and without tinnitus. Further supporting results from a similar study investigating cochlear implant device candidates showed significant improvement from pre-implant SSQ scores to 3 months follow-up [48]. Mean SSQ scores post-BCHA implantation were lower in tinnitus patients, (1.772, SD: 1.920), than patients without tinnitus (2.648, SD: 1.833). This is further supported by a negative correlation (r = −0.231, *p* = 0.141) between tinnitus status and change in SSQ scores post-implantation. SSQ scores from a similar study were significantly lower in cochlear-implanted patients with tinnitus than without [49]. Hearing devices have been shown to improve HRQoL in patients with hearing difficulties and several studies have demonstrated the positive effect of these devices on tinnitus and its psychosocial impact [50,51].

Results from the tinnitus perception assessment pre-and post-implant demonstrated that 29% of patients felt an improvement in tinnitus symptoms post-implant. Comparable findings based on hearing healthcare professionals’ survey results suggested that 60% experienced minor to major relief and 22% of patients experienced major tinnitus relief with hearing aids [52]. Their survey results suggested that 2% of patients felt worse after treatment; however, our study demonstrates that 6% of BCHA patients perceived their tinnitus to be worse after treatment. This falls within the reported range of 0–12% of perceived worse tinnitus annoyance from previous studies [52,53]. The effects on tinnitus perception post-device implantation are not well understood due to the individualistic nature of the disease [36]. In lesser cases, long-standing tinnitus may resist surgical treatment and in others, the act of surgery itself may exacerbate tinnitus symptoms [54]. Although symptoms may be aggravated by hearing loss, the cause of tinnitus could stem from a multitude of health conditions related to muscular disorders or temporomandibular joint and vascular abnormalities [55] and therefore hearing device implantation may not always suppress tinnitus symptoms.

The prevalence of tinnitus among BCHA patients (55%) is considerably higher than the global tinnitus prevalence rate. This can be expected for patients with hearing loss as they are more likely to experience distressing tinnitus symptoms than those able to experience a sound-filled environment [8,22,28]. The prevalence rate in this study is also much higher than observed in previous related findings (35%) [56].

The analysis of two standardised measures in the form of HUI-3 and SSQ questionnaires is a key strength of this study as they are valid instruments used to reliably measure both HRQoL as well as hearing disability [31,32,57]. However, most patients in our sample received transcutaneous devices and were from one geographic location. Therefore, future studies covering a larger proportion of patients who have received percutaneous devices are necessary to determine how percutaneous devices might impact tinnitus. In addition, patients from more diverse geographical locations should be studied to determine whether our findings are applicable to a more diverse patient population. Studies have demonstrated the need for a more comprehensive hearing-attribute subscale design in HUI-3 to improve the HRQoL measure for patients with hearing loss, and this could be useful for future studies in the field [58]. Data on comorbid conditions associated with hearing loss are not included in the study and could influence the HRQoL outcome. Due to the heterogeneous nature of the condition, future studies should include comorbid conditions and further explore the aetiologies of hearing loss to fully appreciate its impact on HRQoL. Tinnitus prevalence increases with age and can be influenced by gender [59] and occupation [60]. This study did not analyse confounders such as age, gender and occupation and their association with the disease. Moreover, this study had a varied age group of between 18 and 66 years old and future research could benefit from a more defined age group for analysis [59]. Furthermore, the simple, non-validated tinnitus questionnaire only provided limited information on tinnitus burden and there is a need for a more detailed and standardised classification system of tinnitus severity in future studies to gain a thorough understanding of potential interventional benefits.

## 5. Conclusions

Hearing loss patients with tinnitus present with a lower HRQoL than patients without tinnitus, however, the improvement in overall HRQoL post-implantation did not vary significantly between these patient groups. Bone conduction hearing implantation improves HRQoL and reduces hearing disability in patients with hearing loss, with or without tinnitus. Furthermore, tinnitus symptoms were improved in over a quarter of tinnitus patients 1 year post-device implantation. These findings demonstrate the importance of hearing rehabilitation in improving the quality of life and hearing disability of patients with or without tinnitus and in providing tinnitus relief in some patients with hearing loss and tinnitus.

## Figures and Tables

**Table 1:** BCHA participants’ demographic data at baseline n = 42.

Participant Characteristics, (n = 42)	n (%)
Age (years) mean ± SD (min–max)	40.21 ± 14.58 (18–66)
Gender	
Female	17 (40.5)
Male	25 (59.5)
Country of residence	
Colombia	36 (85.7)
Poland	6 (14.3)
Tinnitus	
Presence of tinnitus pre implant	23 (54.8)
No presence of tinnitus pre implant	19 (45.2)

**Table 2:** Statistics summary table for bone conduction implant patients with and without tinnitus.

	Tinnitus	Test	*p*-Value	Mean Pre-Implantation (Standard Deviation)	Mean Post-Implantation (Standard Deviation)	Mean Score Improvement (Standard Deviation)	Effect Size (d)/Cohen’s d
**Acoustic Implant: BCHA**							
**HUI-3 Utility Score**	Yes	WSRT	0.218	0.624 (0.310)	0.678 (0.269)	0.054 (0.320)	0.182
	No	WSRT	0.277	0.811 (0.188)	0.876 (0.158)	0.065 (0.224)	0.176
**HUI-3 Hearing Attribute Score**	Yes	WSRT	0.139	0.865 (0.145)	0.916 (0.067)	0.051 (0.147)	0.218
	No	WSRT	0.053	0.857 (0.153)	0.933 (0.096)	0.076 (0.170)	0.313
**Global SSQ Score**	Yes	Paired Samples *t*-test	0.000	4.794 (1.863)	6.566 (1.640)	1.772 (1.920)	0.923
	No	Paired Samples *t*-test	0.000	5.036 (1.729)	7.684 (1.200)	2.648 (1.833)	1.445

**Table 3:** Association between tinnitus status and HRQoL and hearing disability measures in BCHA patients.

	n	HUI-3 Pre-Implant r (*p*-Value)	Change in HUI-3 r (*p*-Value)	Hearing Attribute Pre-Implant r (*p*-Value)	Change in Hearing r (*p*-Value)	SSQ Score Pre-Implant r (*p*-Value)	Change in SSQ Score r (*p*-Value)
Tinnitus status in BCHA patients	42	−0.343 (0.026)	−0.019 (0.906)	0.027 (0.866)	−0.081 (0.612)	−0.069 (0.666)	−0.232 (0.141)

## Data Availability

Aggregated, anonymized study data will be made available upon reasonable request by contacting the corresponding author.
